# Is Augmented Reality Technology Effective in Locating the Apex of Teeth Undergoing Apicoectomy Procedures?

**DOI:** 10.3390/jpm14010073

**Published:** 2024-01-07

**Authors:** Nuria Tamayo-Estebaranz, María José Viñas, Patricia Arrieta-Blanco, Álvaro Zubizarreta-Macho, Juan Manuel Aragoneses-Lamas

**Affiliations:** 1 Department of Medicine and Medical Specialties, Faculty of Health Sciences, Universidad Alcalá de Henares, 28691 Madrid, Spain; nuria.tamayo@edu.uah.es; 2 Faculty of Dentistry, Universidad Alfonso X el Sabio, 28691 Madrid, Spain; mvinapin@uax.es (M.J.V.); parribla@uax.es (P.A.-B.); or jmaragoneses@gmail.es (J.M.A.-L.); 3 Department of Surgery, Faculty of Medicine and Dentistry, University of Salamanca, 37008 Salamanca, Spain; 4 Department of Dentistry, Universidad Federico Henríquez y Carvajal, Santo Domingo 11114, Dominican Republic

**Keywords:** apical location, apicoectomy, augmented reality, cone-beam computed tomography scan, trephine bur

## Abstract

This study seeks to assess the accuracy of apical location using an augmented reality (AR) device with a free-hand method. Sixty (60) osteotomy site preparations were randomly assigned to one of two study groups: A. AR device (AR) (*n* = 30), and B. conventional free-hand method (FHM) (*n* = 30). Preoperative CBCT scans and intraoral scans were taken and uploaded to specialized implant-planning software to virtually plan preparations for the apical location osteotomy sites. The planning software was then used to automatically segment the teeth in each experimental model for their complete visualization using the AR device. A CBCT scan was carried out postoperatively after conducting the apical location procedures. The subsequent datasets were imported into therapeutic software to analyze the coronal, apical, and angular deviations. The Mann–Whitney non-parametric test was used. There were no statistically significant differences identified at the coronal (*p* = 0.1335), apical (*p* = 0.2401), and angular deviations (*p* = 0.4849) between the AR and FHM study groups. The augmented reality technique did not show a statistically significant accuracy of osteotomies for apical location when compared with the conventional free-hand method.

## 1. Background

Augmented reality (AR) is an innovative new technology that involves digital information being overlaid over reality, augmenting the perception of the user [1]. The computer-generated images are superimposed over real-scene images and displayed on computers or other displays [2]. AR is a subtype of virtual reality (VR), which refers to the generation of completely artificial, immersive images that enable real-time interaction within a computer-stimulated environment. With AR, the reality of the user is experienced in real-time, whereas VR entails an imitated reality [3]. Mixed reality (MR) is another version, involving a combination of AR and VR in which the digital and physical worlds are combined [4].

While relatively new, there continues to be development and expansion of the practical uses for these systems within different fields. In the field of medicine, MR and VR are used for teaching and training, and surgical settings often use AR for practice [4]. In this way, surgeons can hone their skills without putting patients at risk, and the learning curve is shorter. Many different fields utilize techniques for overlaying patient information over reality, including urology, neurosurgery, laparoscopic surgery, spinal surgery, hepatobiliary surgery, endoscopic surgery, maxillofacial surgery, catheterizations, pancreatic surgery, and other fields [4,5].

The anatomy involved in craniofacial structures is exceedingly complex, which necessitates sophisticated and highly precise planning prior to surgical interventions. This can be greatly improved through the use of AR technologies, which are also well suited to modern philosophies that favor minimally invasive techniques for maxillofacial surgery [6,7].

The actual operative site in a patient has been successfully supplemented using AR navigation systems, which can display data from a given source as additional graphic information. Other diagnostic tools including X-rays, MRIs, angiograms, and CT scans can be used to make AR integration even more accurate [8,9,10].

Unlike conventional image-guided techniques for surgery, which involve the operator having to glance away from the operative field for information to be visible, AR guidance systems ensure that operators can see information in real-time without averting their gaze from the given surgical field [11,12].

Further computer-generated information is usually superimposed over the surgical field, where it is directly within the line of sight of the operator [13]. A wide array of procedures have been shown to be significantly improved by the use of AR technologies.

Decision making for surgeons can sometimes me made more difficult by extraneous information. By using AR systems during the placement of implants, information can be automatically displayed and limited to whatever is relevant for the surgeon, enabling them to more effectively focus on the current procedure [14,15].

As a result of these benefits, the potential use of AR for other endodontic interventions is under research. When it is not possible to achieve a complete seal using non-surgical orthodontic approaches, periapical surgery is used, removing part of the apex and anatomical complexities through a surgical flap [16]. This prevents the development of microorganisms inside the root by sealing the root canal and removing the apical portion of the root canal. This aims to ensure optimal conditions for the periapical tissue to heal and the insertion device to regenerate [17,18]. The main difficulty is due to the complex anatomy of the pulp–dentin complex, which can lead to periapical surgery being less feasible as a result of impeded access or potential for damaging adjacent anatomical structures. In these cases, AR technologies provide an alternative for achieving more predictable and successful surgical results.

This study assessed the accuracy of apical location using an AR device with a free-hand method. The null hypothesis (H_0_) is as follows: there is no difference in accuracy between the AR technique and the free-hand technique.

## 2. Methods

### 2.1. Study Design

A total of 84 upper teeth representative of all sectors, removed due to orthodontic and periodontal concerns, were treated between November 2021 and March 2022 at the Alfonso X El Sabio University (Spain). A preliminary study was used to analyze the sample size [19], with an effect size of 87.2 (greater than 80 was considered adequate). A total of 60 preparations of osteotomy sites were studied to obtain an effect of 80.00% for determining which differences were statistically significant. The bilateral Student’s *t*-test was used to assess the null hypothesis, with a significance of 5.00%. The ensuing article was written in accordance with the principles established by the Preferred Reporting Items for Laboratory Studies in Endodontology (PRILE) [20,21].

### 2.2. Experimental Procedure

Fourteen of the teeth selected for study were inserted in six different epoxy resin materials (Ref. 20-8130-128, EpoxiCure^®^, Buehler, IL, USA). Sixty preparations of osteotomy sites were randomly assigned (Epidat 4.1, Galicia, Spain): A. AR device (Hololens2, Redmond, WA, USA) (AR) (*n* = 30), and B. free-hand method (FHM) (*n* = 30). All teeth were analogous in anatomy. The location of the teeth was reproduced by a silicone splint so that they had the same apical position. This silicone was printed with the traditional method using acrylic resin and a dental training model, followed by subsequent placement of the teeth. The epoxy resin was then mixed in accordance with manufacturer recommendations. Preparations of the osteotomy sites were carried out in apical locations that had been randomly chosen (Epidat 4.1, Galicia, Spain).

**Figure 1:**
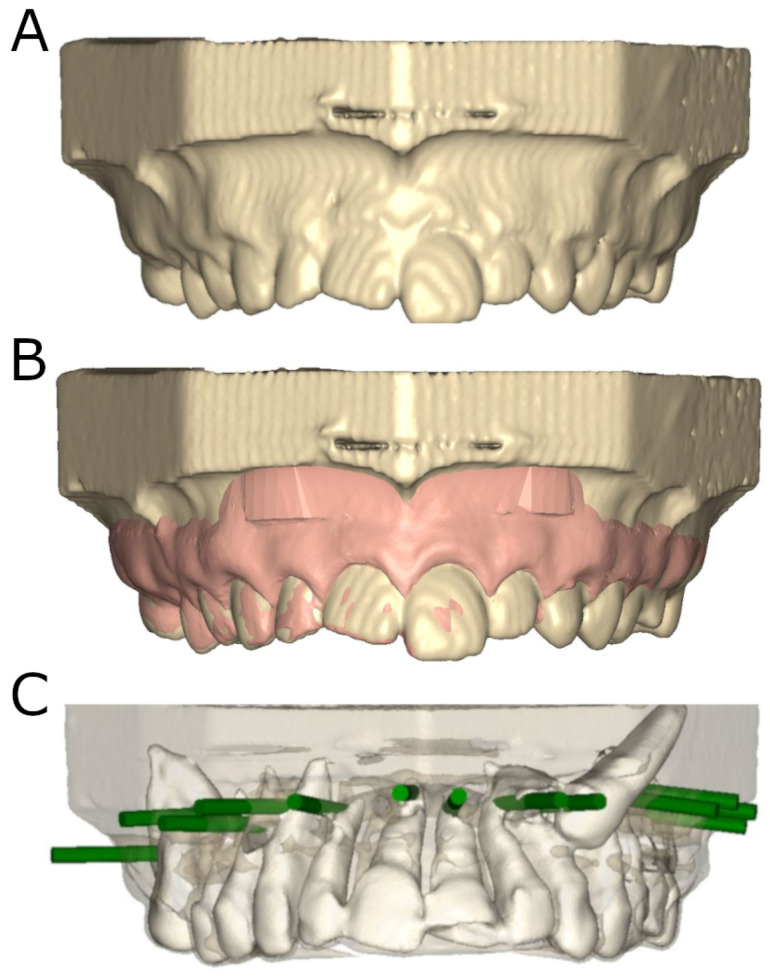
(**A**) Image from CBCT scan, (**B**) alignment of CBCT scans with STL digital files, (**C**) front view of virtually planned preparations of osteotomy sites (green cylinders), with surrounding tissues.

A cone-beam computed tomography scan (CBCT) (WhiteFox, Acteón Médico-Dental Ibérica S.A.U.-Satelec, Merignac, France) was performed before the therapeutic procedures (8.0 mA, 7.20 s, 105.0 kV peak, and a 15 × 13 mm^2^ field of view). A digital impression was performed through a 3D intraoral scan (True Definition, 3M ESPE™, Saint Paul, MN, USA). The resulting datasets were managed in therapeutic planning software (NemoScan^®^, Nemotec, Madrid, Spain) so that the preparations of the osteotomy sites for apical location could be planned. This virtual planning used a diameter of 3.5 mm and length of 13.0 mm. Data from the CBCT and 3D surface scans were aligned, overlaying anatomical points of reference located at the crowns of the teeth. Virtual planning of the preparations of osteotomy sites were carried out at an angle of 90° with respect to the longitudinal axes of the teeth at the apex of each tooth [22] (Figure 1).

**Figure 2:**
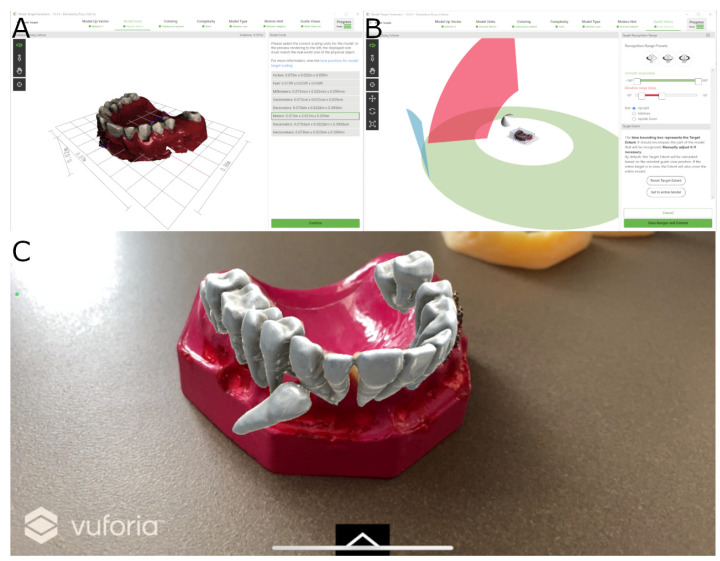
(**A**,**B**) AR device software planning process and (**C**) STL digital file illustration of the segmented teeth virtually aligned with the experimental model.

Therapeutic planning software was then used to automatically segment the teeth in each experimental model so that the operator could fully visualize the root and crown of each tooth. Lastly, the STL digital file was imported into the AR device for apical location in all space planes (INNOAREA, Valencia, Spain) in the AR study group (Figure 2A–C).

An individual operator with 10 years of experience prepared the osteotomies using a trephine bur (Ref.: 330205486001, Antarctica, Pleumeleuc, France) at 100,000 rpm under irrigation.

### 2.3. Measurement Procedure

**Figure 3:**
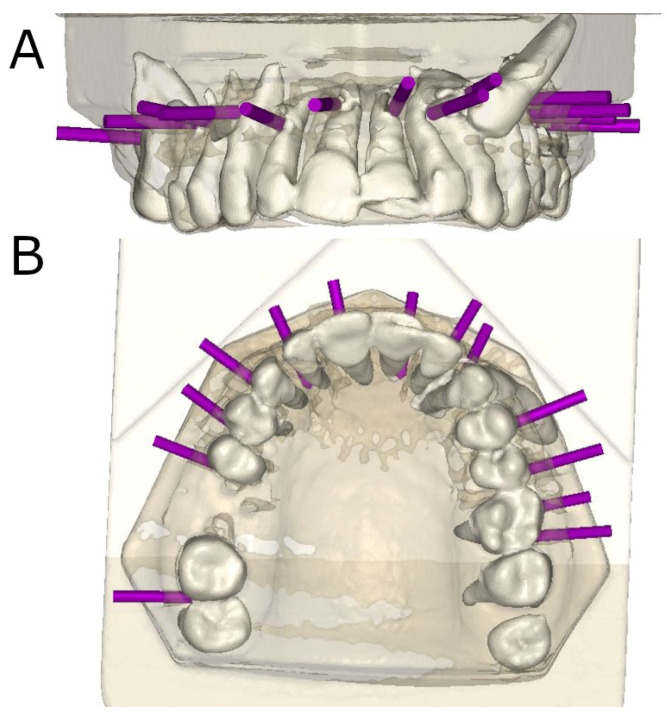
(**A**) Front view and (**B**) occlusal view of the postoperatively rendered CBCT scan with the preparations of the osteotomy sites (purple cylinders).

CBCT of the models was performed after preparations of the osteotomy sites for apical location (Figure 3A,B).

**Figure 4:**
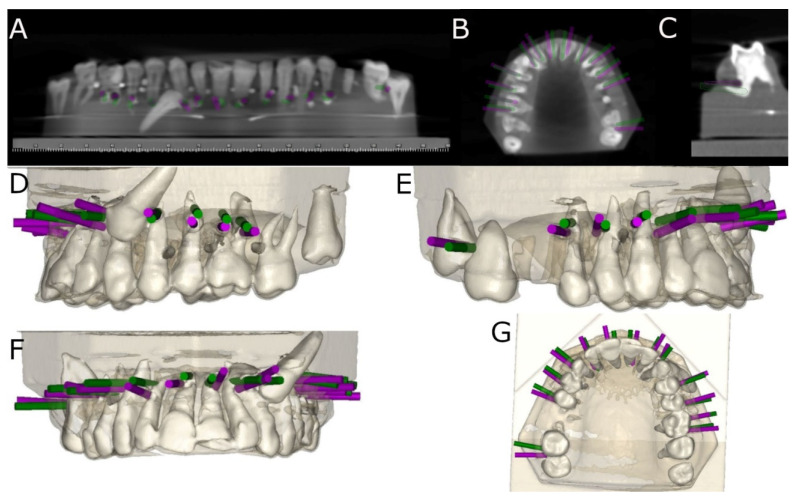
(**A**) Coronal view, (**B**) occlusal view, and (**C**) sagittal view of the CBCT scan of the procedure for analyzing deviations between planned (green cylinder) and performed (pink cylinder) preparations of osteotomy sites. (**D**) Left lateral, (**E**) right lateral, (**F**) frontal, and (**G**) occlusal view of the postoperatively rendered CBCT scan with the preoperatively planned preparations of osteotomy sites (green cylinders) and the postoperatively performed preparations of osteotomy sites (purple cylinders).

The postoperative CBCT scans and virtually planned osteotomy site preparations were managed in therapeutic planning software. The data were superimposed to determine the angle (center of the cylinder) and horizontal deviation (apical end-point and coronal entry-point). A different observer performed the measurements (Figure 4A–G).

### 2.4. Statistical Tests

For statistical analysis, the relevant variables were added to SPSS 22.00 for Windows. The results are expressed as mean and standard deviation (SD) for quantitative variables. The Mann–Whitney test was used to measure the difference between the AR and FHM in mean deviation in the planned and performed preparations of osteotomies with *p* < 0.05.

## 3. Results

Table 1 displays the mean and SD values of the coronal (mm), apical (mm), and angular (°) deviations in the osteotomy preparations for each study group.

**Figure 5:**
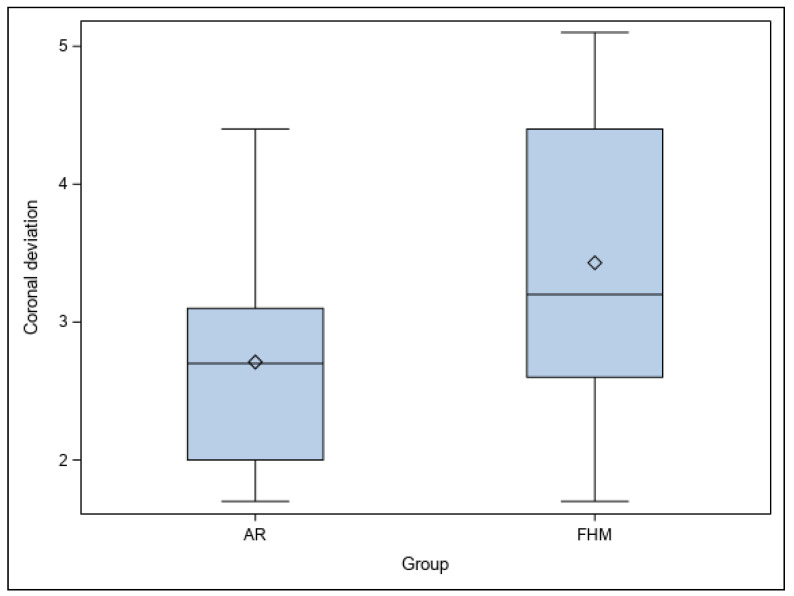
Box plot of coronal deviations of AR and FHM study groups. “◊” represent the median.

No statistically significant differences were shown between the planned and performed osteotomy site preparations of the study groups at the coronal level (*p* = 0.1335) (Figure 5).

**Figure 6:**
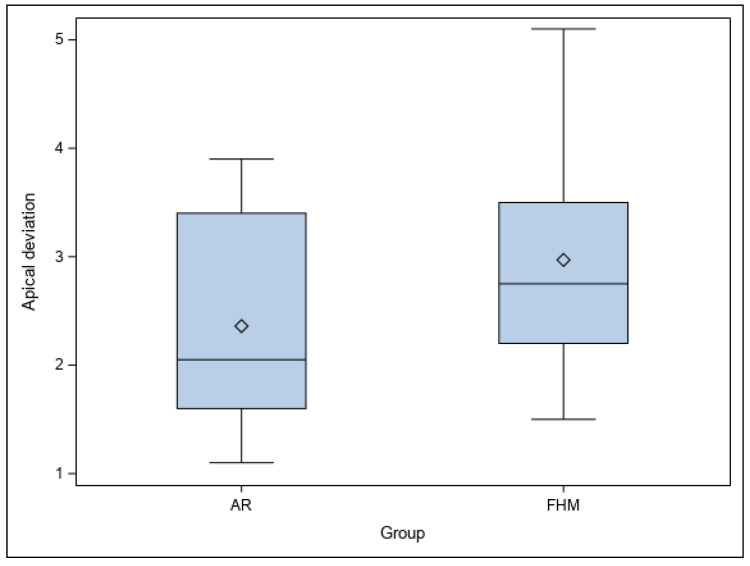
Box plot of apical deviations of AR and FHM study groups between planned and performed osteotomy site preparations for apical location. “◊” represent the median.

Similarly, no statistically significant differences were shown between the planned and performed osteotomies of the study groups at the apical level (*p* = 0.2401) (Figure 6).

**Figure 7:**
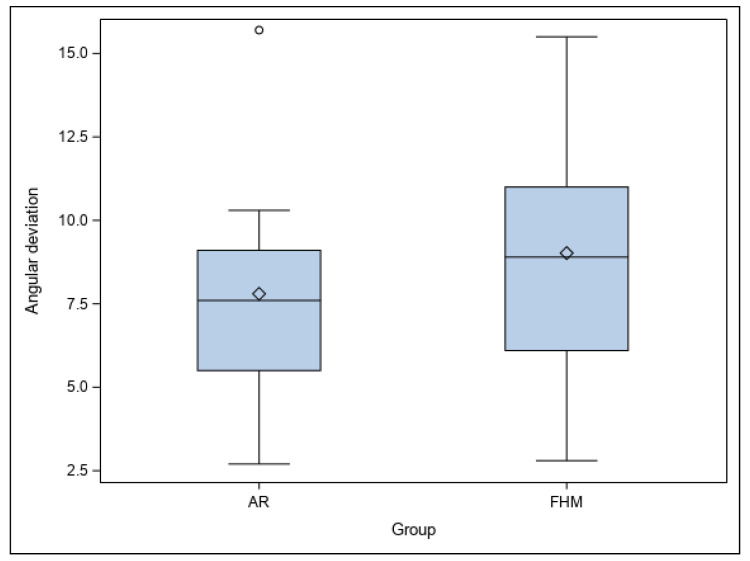
Box plot of angular deviations of AR and FHM study groups. “◊” represent the median. “◦” represent an extreme value.

Lastly, no statistically significant differences were shown between the planned and performed osteotomies of the study groups at the angular level (*p* = 0.4849) (Figure 7).

## 4. Discussion

The results confirmed the null hypothesis (H_0_) that the AR and free-hand techniques are equally accurate for apical location.

It remains unclear how these techniques originated. Cabero and Barroso trace their beginnings to a Sensorama system for film projection from 1962, an iconic experience involving the addition of 3D stereoscopic vision [1]. Boeing researcher Caudell first coined the term “AR” in 1990; together with Mizell, in 1992, he developed a military combat aircraft prototype that could project images of planes onto a surface [3]. After smartphones and tablets were developed in 2013, Google launched Google Glass, having developed an HMD with a voice interface and hands-free system the user could use to send text messages, browse the internet, and make calls. The Hololens was introduced by Microsoft in 2015, enabling users to use their voice, gaze, and gestures to see and interact with 3D holographic 3D virtual objects [2].

While other medical fields share much in common with dentistry, there are also many things that make it unique. Dentistry is yet another field in which outcomes for diagnosis, treatment, and education can be improved with the successful implementation of technologies using AR [23]. A dental implant positioning system was introduced in 1995, which enabled suggested positions to be projected over the patient [24].

A retinal imaging screen was implemented in AR technologies for use in the placement of surgical implants, a procedure in which the oral surgical site is very small and glancing away to look at the monitor poses risks.

The results indicate that the AR technique did not show statistically significant, accurate apical location at the coronal, apical, and angular level compared to the FHM technique.

This measurement methodology has been supported through studies of the precision of dental implant placement, which have observed coronal deviations of 0.99 mm, apical deviations of 1.24 mm, and angular deviations of 3.81 mm [25]. These promising results encouraged researchers to employ image-guided navigation techniques in other dental disciplines such as endodontics, with statistically significant differences reported at the coronal (*p* < 0.0001), apical (*p* < 0.0001), and angular (*p* < 0.0001) levels [19]. Accuracy is of the utmost importance for both apical and root canal location given the small working field and the fact that there is a greater risk of intraoperative complications with these procedures. Several studies have examined whether or not computer-aided navigation techniques provide the greatest accuracy. When used for apical location of the root apex, conservative surgical access cavities enable greater accuracy and reduced patient discomfort after procedures, improved periapical healing of bone defects, and reduced operating time, as well as avoiding any additional risks of damage to adjacent structures [26]. Therefore, clinicians should consider drilling using computer-aided static navigation techniques, especially when there is reduced surgical access; these techniques carry less risk of cortical loss and periapical tissue damage despite the difficulty in inserting and maneuvering ultrasonic tips along the longitudinal axis of the tooth and the limited visibility of resected roots [27]. However, the higher costs, steep learning curves, longer times, and lack of accuracy of computer-aided navigation techniques may lead clinicians to eschew these techniques in favor of traditional free-hand techniques for surgical procedures.

When carrying out microsurgical endodontic procedures, locating the root apex is a challenge for clinicians [28]. Traditionally, these procedures use illumination, CBCT scans, microinstruments, and magnification to improve the success rates of root apex location, but computer-assisted static navigation techniques may be up to 27 times more successful at root apex location than conventional microsurgical procedures [29]. Furthermore, computer-assisted static navigation techniques for apical location procedures have a 96.8% success rate (confidence interval of 93.0% to 100%), which makes them highly recommended for apical root location during microsurgical endodontic procedures. Trephine burs are generally used in planned techniques for apical root location using computer-assisted static navigation techniques, as their cylindrical shape makes undesirable deviations less likely during drilling. That being said, if the root apex is not located using computer-aided navigation techniques, or if the conditions are not favorable for root-end cavity preparation or root apex resection during osteotomy preparation, then the latter must be conducted using conventional free-hand techniques with PUI, which allows clinicians to adjust the direction of the osteotomy preparation in a more conservative manner than the trephine bur technique. These conventional free-hand techniques are especially recommended in cases of limited mouth openings, or posterior region procedures in which insertion of the surgical splint proves difficult [30,31].

Be that as it may, there is no such thing as a success rate of 100%, and the accuracy of new technologies for root apex location is always under study. Gambarini et al. described a clinical case in which apical location during endodontic microsurgery was carried out using computer-assisted dynamic navigation techniques. These techniques use stereoscopic motion-tracking cameras within an optical triangulation tracking system to guide the drilling process in real-time, which enables the clinician to achieve the pre-planned osteotomy trajectory, angle, and depth [3]. They are frequently used for dental implant placement, and studies indicate that they result in statistically significant, lower deviation values (*p* ˂ 0.05) at the coronal entry point (0.71 ± 0.40 mm), apical end point (1.00 ± 0.49 mm), and angular deviation (2.26 ± 1.62°) level when compared with traditional free-hand techniques for dental implant placement [32,33]. The field of endodontics also uses computer-aided dynamic navigation techniques to prevent complications and enable more accurate root canal location [34,35,36]. On the other hand, a recent systematic review and meta-analysis did not find statistically significant differences in the success rates of root canal location between static and dynamic computer-aided navigation techniques (*p* = 0.185) [37]. There were statistically significant differences between computer-aided static navigation techniques and conventional free-hand techniques in apical location (*p* < 0.0001) [4]. Despite this, traditional free-hand techniques are still widely used, and several articles reference the success rates of apical location using trephine bur devices and piezoelectric ultrasonic inserts [38,39,40,41]. Further study is needed to corroborate findings regarding the accuracy of apical location when using conventional free-hand techniques.

This in vitro study was limited in scope due to its experimental nature; a real-life clinical situation would likely not have the same level of similarities in tooth anatomy and position. Regardless, the teeth were selected based on their anatomy, as well as being randomized. The silicone splint was used to ensure repeatability of the dental position in all experimental models. The methodology of this study is easily applicable to clinical studies.

## 5. Conclusions

The results indicate that the free-hand and the AR techniques do not show a statistically significant accuracy for apical location.

## Figures and Tables

**Table 1:** Mean and SD values for the coronal (mm), apical (mm), and angular (°) deviations in the osteotomies.

Location	Group	*n*	Mean	SD	Minimum	Maximum
Coronal	AR	30	2.71 ^a^	0.79	1.70	4.40
	FHM	30	3.43 ^a^	1.21	1.70	5.10
Apical	AR	30	2.36 ^a^	0.96	1.10	3.90
	FHM	30	2.97 ^a^	1.26	1.50	5.10
Angular	AR	30	7.80 ^a^	3.57	2.70	15.70
	FHM	30	9.02 ^a^	4.07	2.80	15.50

^a^ (*p* < 0.05).

## Data Availability

The datasets generated and/or analyzed during the current study are not publicly available, due to the involvement of personal data of patients, but they are available from the corresponding author on reasonable request.
